# From metabolic substrate to epigenetic regulation: roles and mechanisms of lactylation in brain health and disease

**DOI:** 10.3389/fnmol.2026.1779468

**Published:** 2026-04-08

**Authors:** Wei Chen, Ruobing Li, Jinlong Zhang, Jun Lei, Xicheng Jiang, Xiaowei Sun

**Affiliations:** 1 College of Basic Medicine, Heilongjiang University of Chinese Medicine, Harbin, Heilongjiang, China; 2 Rehabilitation Department, Heilongjiang Provincial Hospital of Traditional Chinese Medicine, Harbin, Heilongjiang, China; 3 Rehabilitation Department II, First Affiliated Hospital of Heilongjiang University of Chinese Medicine, Harbin, Heilongjiang, China

**Keywords:** epigenetic regulation, lactate, lactate metabolism, neurological disorders, protein lactylation

## Abstract

Lactate, traditionally regarded as a metabolic byproduct of glycolysis, is now recognized as a critical signaling molecule in the central nervous system. Emerging evidence indicates that lactate participates in a dynamic metabolic–epigenetic regulatory network through protein lactylation, a post-translational modification capable of modulating chromatin structure and gene transcription. We summarize the physiological roles of lactate in neuronal–glial metabolic coupling and highlight cell-type-specific functions of the lactate–lactylation axis under both normal and pathological conditions. Particular emphasis is placed on its involvement in ischemic stroke, neurodegenerative diseases such as Alzheimer’s disease and Parkinson’s disease. Available findings indicate that this axis is integral to synaptic plasticity, neuroinflammatory balance, and metabolic homeostasis. Under pathological conditions, excessive lactate accumulation promotes aberrant lactylation patterns that may drive persistent inflammation, metabolic reprogramming, and neuronal dysfunction by reshaping chromatin accessibility and transcriptional landscapes. Collectively, the lactate–lactylation axis represents a unifying mechanistic framework linking metabolic flux to epigenetic regulation in neurological disorders and may serve as a promising source of diagnostic biomarkers and precision therapeutic targets.

## 1 Introduction

For a long time, lactate was regarded as a classical by-product of glucose metabolism that accumulated primarily under hypoxic conditions and was associated with muscle fatigue and metabolic stress (Cheung et al., 2003; Mintun et al., 2004; Haas et al., 2016; Ferguson et al., 2018). In this traditional view, glucose-derived pyruvate entered the tricarboxylic acid (TCA) cycle under sufficient oxygen supply, whereas lactate formation was considered a metabolic endpoint of anaerobic glycolysis (Schirinzi et al., 2020; Fantin et al., 2006). Consequently, lactate was long perceived as metabolic waste rather than a biologically active molecule.

This concept has been fundamentally revised with advances in metabolic research. Accumulating evidence demonstrates that lactate is continuously produced under aerobic conditions and functions as a key metabolic intermediate linking glycolysis and oxidative metabolism (Mintun et al., 2004; Schirinzi et al., 2020; Fantin et al., 2006). Lactate can be reutilized as a carbon source for the TCA cycle and serves as an important substrate for energy production in multiple tissues, including the brain. Beyond its metabolic role, lactate has emerged as a signaling molecule that participates in the regulation of cellular activity and physiological homeostasis (Wang et al., 2023; Feng et al., 2022).

In the central nervous system, lactate plays a particularly prominent role. The brain is a highly energy-demanding organ and relies on precise metabolic coordination among neurons, astrocytes, microglia, oligodendrocytes, and the blood–brain barrier. Lactate contributes not only to neuronal energy supply but also to neuromodulation, neurovascular coupling, and protection against excitotoxic injury. Activation of hydroxycarboxylic acid receptor 1 (HCAR1), which is highly expressed in the brain, mediates lactate-dependent signaling pathways that regulate neuronal survival and metabolic adaptation (Yao S. et al., 2023). These findings establish lactate as an essential regulator of brain function rather than a passive metabolic by-product.

A major conceptual advance in lactate biology was the discovery of protein lactylation, a novel post-translational modification directly derived from lactate metabolism (Vander Heiden et al., 2009; Wei et al., 2024). Under conditions of enhanced glycolytic flux or metabolic stress, intracellular lactate accumulation is accompanied by a marked increase in lysine lactylation. This modification is dynamic and reversible and can alter protein structure, surface charge, and molecular interactions, thereby influencing protein stability, enzymatic activity, and transcriptional regulation (Mintun et al., 2004; Wei et al., 2024).

Emerging evidence indicates that protein lactylation is closely linked to neuronal activity and stress-related responses in the brain. Neuronal excitation, social stress, and altered metabolic states have been shown to modulate lactylation levels in neural tissues (Hagihara et al., 2021). Excessive lactate uptake by neurons can increase reactive oxygen species production, impair mitochondrial function, and exacerbate oxidative stress, ultimately contributing to axonal degeneration and neuronal dysfunction (Jia et al., 2021; Scavuzzo et al., 2020). These findings suggest that dysregulated lactate metabolism and aberrant lactylation may form a pathogenic axis in neurological disorders.

**Figure 1:**
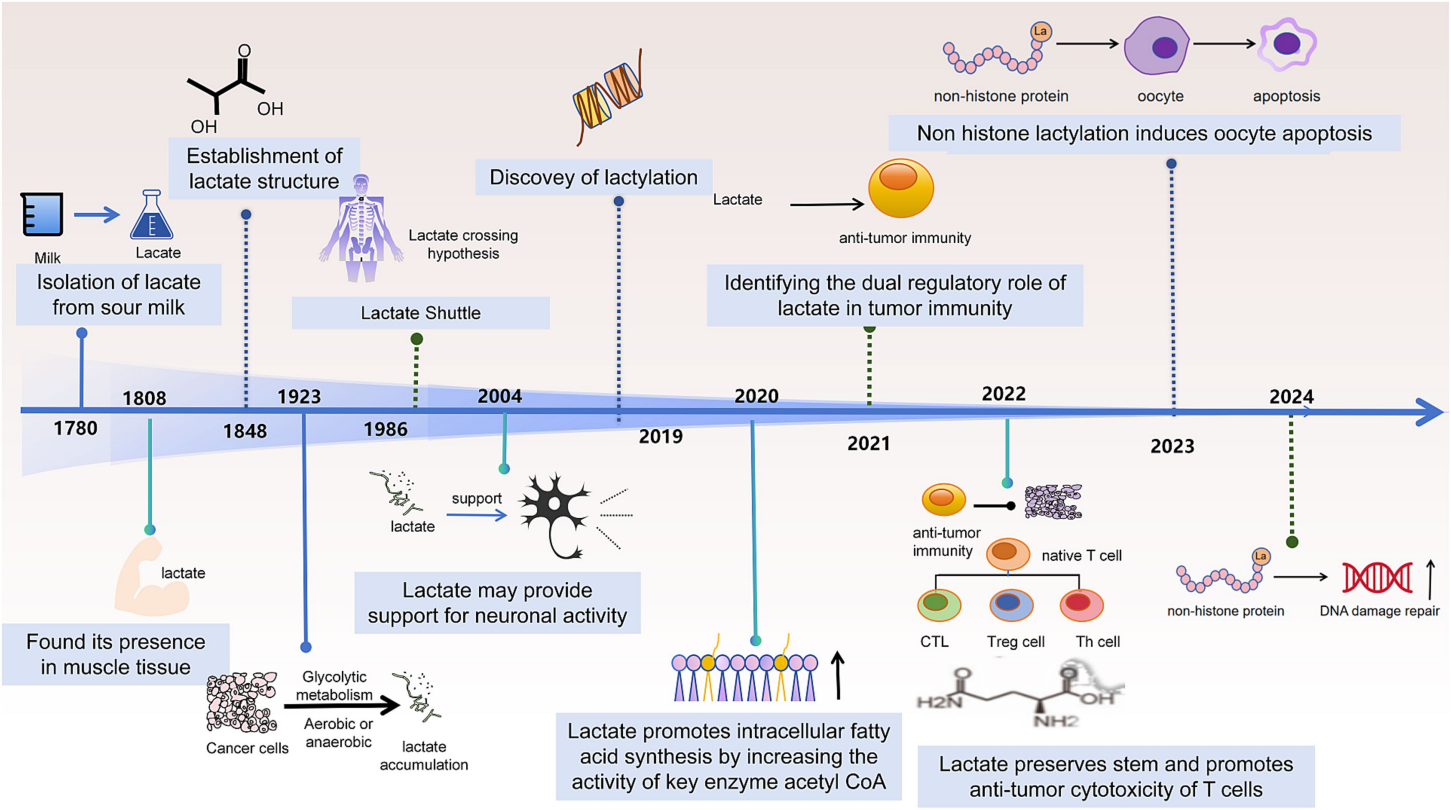
Evolutionary journey of lactate’s biological functions. Beginning with the isolation of lactate in 1780 and its structural elucidation in 1848, lactate has gradually evolved from a simple metabolite to a pivotal signaling molecule. Key milestones include the proposal of the lactate shuttle hypothesis (1923), recognition of its supportive role in neuronal activity (1986), and the groundbreaking discovery of lactate modification (2019). Subsequent decades of research have revealed lactate’s diverse roles in lipid metabolism, bidirectional regulation of tumor immunity, non-histone lactate modification-induced cell death, as well as in DNA damage repair and stem cell biology.

Current studies indicate that lactate is a multifunctional metabolite that integrates energy metabolism, cellular signaling, and epigenetic regulation in the nervous system. Protein lactylation provides a direct molecular mechanism linking metabolic states to gene expression and cellular behavior. Understanding how lactate metabolism and lactylation are regulated in the brain, and how their dysregulation contributes to neurological disease, has therefore become an important topic in contemporary neuroscience research (Figure 1).

A major conceptual advance in lactate biology was the discovery of protein lactylation, a novel post-translational modification directly derived from lactate metabolism (Vander Heiden et al., 2009). Under conditions of enhanced glycolytic flux or metabolic stress, intracellular lactate accumulation is accompanied by a marked increase in lysine lactylation. This modification is dynamic and reversible and can alter protein structure, surface charge, and molecular interactions, thereby influencing protein stability, enzymatic activity, and transcriptional regulation (Certo et al., 2021).

Emerging evidence indicates that protein lactylation is closely linked to neuronal activity and stress-related responses in the brain. Neuronal excitation, social stress, and altered metabolic states have been shown to modulate lactylation levels in neural tissues (Reuter et al., 1980). Excessive lactate uptake by neurons can increase reactive oxygen species production, impair mitochondrial function, and exacerbate oxidative stress, ultimately contributing to axonal degeneration and neuronal dysfunction (Yang D. et al., 2022; Chen et al., 2021). These findings suggest that dysregulated lactate metabolism and aberrant lactylation may form a pathogenic axis in neurological disorders.

Collectively, current studies indicate that lactate is a multifunctional metabolite that integrates energy metabolism, cellular signaling, and epigenetic regulation in the nervous system. Protein lactylation provides a direct molecular mechanism linking metabolic states to gene expression and cellular behavior. Understanding how lactate metabolism and lactylation are regulated in the brain, and how their dysregulation contributes to neurological disease, has therefore become an important topic in contemporary neuroscience research.

In this review, we summarize current advances in lactate metabolism and protein lactylation in the central nervous system. We focus on their molecular mechanisms, regulatory pathways, and cell-type-specific roles, and discuss emerging evidence linking the lactate–lactylation axis to major neurological disorders, including ischemic stroke, neurodegenerative diseases, epilepsy, psychiatric disorders, and glioma.

## 2 Lactate metabolism in the brain

### 2.1 Lactate production in the brain

The brain is a highly energy-demanding organ and consumes about one-fifth of the body’s total energy (Belanger et al., 2011). Under normal conditions, glucose is its primary fuel. However, in several specific situations, lactate serves as an efficient alternative energy source and plays a critical role. It also influences brain function through dedicated receptors (Proia et al., 2016; Wang Y. et al., 2022). Lactate affects neuronal excitability, pH regulation, fluid balance, neurovascular coupling, and long-term memory formation (Dienel, 2019). At rest, the brain accounts for 13% of total lactate production and 8% of its utilization. In addition, when neuronal activity in certain brain regions becomes extremely high, and the energy demand exceeds the rate of aerobic glucose oxidation, neurons rapidly take up and use lactate. This process helps maintain their normal function (Wang Y. et al., 2024; Smith et al., 2003; Van Hall, 2010). In the brain, lactate is mainly synthesized from glucose through glycolysis. This process occurs in several cell types, especially in glial cells. Astrocytes are the most representative. As central participants in the astrocyte–neuron lactate shuttle (ANLS), they serve as the major site of glycolysis (Allen and Lyons, 2018). Astrocytes take up glutamate at synapses, triggering aerobic glycolysis. This increases glucose uptake and drives lactate release. Neurons then receive and use this lactate as a preferred energy substrate (Dienel, 2019). Extensive studies show that glycolytic activity in astrocytes increases in regions such as the hippocampus and prefrontal cortex during complex cognitive tasks. Lactate production in these surrounding astrocytes rises significantly (Falkowska et al., 2015; Yang et al., 2014).

In the brain, the physiologically normal lactate concentration is 2–5 mM, nerve cells can expand to 4–10 mM during activity, at which level lactate is oxidized via the ANLS pathway to generate energy (Yao S. et al., 2023). When the lactate concentration in the brain exceeds 10 mM, lactate induces aberrant histone lactylation (e.g., overactivation of H3K18la) (Han et al., 2023). Excessive lactate accumulation may contribute to neuroinflammation and oxidative stress in the nervous system (Ran et al., 2026). At 50 mM, it completely reverses the anti-inflammatory phenotype of microglia, promotes the release of pro-inflammatory factors, and induces neural damage (Zhang Y. et al., 2025).

### 2.2 Lactate transport in the brain

Lactate is a hydroxycarboxylic acid. Recent studies show that it exists in three common isomeric forms: D-lactate, L-lactate, and DL-lactate (Rabinowitz and Enerback, 2020). In the human body, lactate mainly appears as the two optical isomers L-lactate and D-lactate. D-lactate is the optical isomer of L-lactate. L-lactate is far more abundant in humans and other mammals. It is also the dominant form in nature. L-lactate is produced directly through glycolysis, is present in most cell types, and plays a central role in energy metabolism and signaling (Lu et al., 2025). D-lactate arises through several sources, including diet, gut bacteria, the metabolism of propylene glycol, and the methylglyoxal pathway (Xin et al., 2022; Monroe et al., 2019). In the glyoxalase pathway, a branch of glycolysis, glucose is first converted to methylglyoxal. Methylglyoxal is then processed by glyoxalase 1 (GLO1) and glyoxalase 2 (GLO2) to generate D-lactate together with glutathione. Gut microbes such as Lactobacillus species can also convert glucose directly into D-lactate, which then enters systemic circulation (Lu et al., 2025). Under normal physiological conditions, the concentration of L-lactate in blood is roughly 100 times higher than that of D-lactate. D-lactate remains at much lower levels compared with L-lactate (Moreno-Yruela et al., 2022). In the brain, L-lactate is maintained at 0.8–2.0 mM, while D-lactate remains below 0.02 mM (Moreno-Yruela et al., 2022). Biochemically, L-lactate is endogenously produced through glycolysis and serves as the primary oxidative fuel for neurons and the substrate for enzymatic L-lactylation (Lu et al., 2025). D-lactate is a secondary byproduct of the glyoxylate pathway and gut microbial metabolism, lacking mitochondrial oxidizing capacity in the central nervous system and undergoing non-enzymatic D-lactylation solely mediated by lactylglutathione (Xin et al., 2022; Monroe et al., 2019). L-lactate is a critical substrate for neuronal oxidative metabolism, promoting protein synthesis, enhancing synaptic remodeling, and increasing axonal excitability during learning and memory formation (Levitt and Levitt, 2020). Abnormal elevation of D-lactate may lead to various neurological symptoms (Kang et al., 2006). When local L-lactate concentrations persistently exceed physiological thresholds or when D-lactate levels abnormally rise due to methylglyoxal accumulation, and this elevated state persists beyond the acute stress window (Magistretti and Allaman, 2018), excessive histone lactylation modifications evolve from physiological transcriptional fine-tuning into irreversible pathological remodeling. This results in genomic instability and pro-inflammatory phenotype locking, transforming lactate from a neuroprotective energy source into a pathogenic factor driving neuronal damage (Zhang et al., 2019).

L-lactate and D-lactate are transported into and out of cells by the monocarboxylate transporter (MCT) family (Enerson and Drewes, 2003). This process regulates intracellular lactate levels. Three major MCT subtypes are involved: MCT1, MCT2, and MCT4. In the brain, MCT1 is widely expressed in astrocytes, endothelial cells, and neurons, and supports bidirectional transport of lactate and pyruvate (Zhang L. et al., 2024). MCT2 is mainly expressed in neurons, especially in synaptic regions, and facilitates neuronal uptake of lactate and other metabolites (Medel et al., 2022). MCT4 is expressed primarily in glial cells, particularly astrocytes, and exports lactate from cells. This export supports intracellular pH balance and energy metabolism (Ma et al., 2024). MCT4 drives L-lactate efflux, while MCT1 and MCT2 mediate lactate influx (Broer et al., 1998, 1999; Wilson et al., 1998). The affinity of each subtype for lactate differs. High-affinity transporters take up extracellular lactate even when intracellular levels are low. Low-affinity transporters export lactate and help reduce intracellular concentrations (Jha and Morrison, 2020). Under normal physiological conditions, brain lactate remains relatively stable. MCT1 shows the highest affinity, and its expression fluctuates with lactate levels (Philips et al., 2021). MCT2 has intermediate affinity. MCT4 has the lowest affinity (Philips and Rothstein, 2017).

Lactate signaling depends on MCT-mediated transport. This process is essential not only for energy metabolism but also for metabolite exchange among different brain cell types. These exchanges are necessary for maintaining normal physiological functions. In cells with high glycolytic activity, MCT1 supports lactate export, which relies on the lactate concentration gradient across the membrane (Medel et al., 2022). By transporting lactate, MCT1 promotes metabolic exchange and enhances neuronal metabolism (Saab and Nave, 2016; Li R. et al., 2024; Zhao et al., 2020). Reduced MCT1 expression causes severe neuronal damage and suppresses axonal regeneration (Philips et al., 2021). MCT2 is mainly found in neurons and endothelial cells. It interacts with the lactate receptor HCAR1 and modulates downstream signaling (such as reducing cAMP production). Through this mechanism, it improves glycolysis and reduces lactate production, helping regulate energy metabolism (Arnsten et al., 2012). Overexpression of MCT2 in a rat stroke model enhances mitochondrial biogenesis. This increase in mitochondrial number and function improves cellular energy metabolism and ultimately enhances cognitive performance in the rats (Yu X. et al., 2021). MCT4 is expressed only in astrocytes, vascular endothelial cells, and microglia (Proia et al., 2016; Cantando et al., 2024). Studies show that increased expression of key glycolytic enzymes promotes lactate production, while upregulation of MCT4 helps establish transcellular lactate shuttling (Yamagata, 2022; Rosafio et al., 2016). Elevated MCT4 expression is associated with impaired neurite growth and increased apoptosis (Hong et al., 2020). Within the brain, neurons metabolize lactate produced by astrocytes, while astrocytes take up glutamate released by neurons (Brooks, 2018). After L-lactate enters neurons through MCT2, it enhances neuronal excitability by modulating intracellular signaling pathways. This activation increases glycolytic activity in astrocytes. Lactate exported by astrocytes through MCT4 then becomes available for neuronal uptake through MCT2, serving as an energy substrate for neurons.

Studies show that MCTs promote the uptake of extracellular lactate by macrophages, leading to lactylation of HMGB1. Inhibiting MCTs blocks this process and suppresses lactate-induced lactylation under high-lactate conditions (Yang K. et al., 2022). Direct lactylation is also regulated by specific enzymes, so enzyme activity plays a key role in this modification (Moreno-Yruela et al., 2022). Neural activity may further influence lactylation. In mice subjected to social defeat stress, brain lactate levels and lactylation correlate positively with neuronal activity, suggesting that lactylation contributes to neural regulation in depression and anxiety (Hagihara et al., 2021). Proteins carry multiple types of post-translational modifications, and these modifications can interact. This phenomenon, known as crosstalk, is another important factor affecting lactylation. Lactylation and acetylation, for example, compete for lysine residues on histone H3 (Hagihara et al., 2021). The two enzymes primarily act on the same amino groups of lysine residues and share partial acyltransferase activity, thereby creating substrate competition within cells. When glycolysis becomes active and leads to lactate accumulation, lactylation competes with acetylation as a “late metabolic clock”. This interaction enables cells to transition smoothly from an initially acetylation-driven pro-inflammatory response to a lactylation-driven homeostatic repair gene expression, achieving precise temporal regulation of gene transcription patterns through metabolic flux (Hagihara et al., 2021). In addition, lactylation of METTL16 promotes m6A modification of FDX1 mRNA, increases FDX1 protein levels, and drives copper-dependent cell death in gastric cancer (Sun et al., 2023). Histone H3K18 lactylation and upregulation of METTL3 and m6A levels in the mouse brain, along with increased LCN2 protein levels, activated astrocytes to undergo pro-inflammatory type A1 transformation (Gao et al., 2025). Lactylation is a complex process shaped by multiple regulatory inputs. The blood–brain barrier (BBB) also contributes to lactate homeostasis. Its specialized structure and function allow selective control of circulating lactate, helping maintain dynamic lactate balance in the brain. Under physiological conditions, MCTs at the BBB tightly regulate lactate transport and sustain a stable concentration gradient between the brain and peripheral blood. When peripheral lactate rises due to exercise or metabolic disturbances, the BBB adjusts MCT expression or activity and increases lactate uptake into the brain (Wang Y. et al., 2022). When lactate becomes excessive in the brain, the BBB enhances lactate efflux to prevent harmful accumulation.

### 2.3 Clearance of lactate in the brain

Under normal physiological conditions, even in the presence of adequate oxygen, increased neuronal activity stimulates nearby astrocytes to take up glucose. During neuronal firing, neurotransmitters such as glutamate are released. Astrocytes absorb glutamate through glutamate transporters on their membranes. This uptake activates a series of metabolic responses inside astrocytes, including an increase in glucose uptake. GLUT1 is the main transporter responsible for this process (Hu et al., 2021). Once inside the cell, glucose rapidly enters the glycolytic pathway. Through the actions of key enzymes—hexokinase, phosphofructokinase-1, and pyruvate kinase—it is gradually broken down into pyruvate (Wu et al., 2023). Under sufficient oxygen, pyruvate would normally enter mitochondria for further oxidation in the tricarboxylic acid cycle. However, when the brain is in a state of high metabolic demand, such as during learning or memory processing, pyruvate is instead reduced to lactate by lactate dehydrogenase A (LDHA) using hydrogen supplied by NADH. Studies indicate that lactate is not only the end product of glycolysis. It can also serve as an energy substrate for highly active neurons or act through a lactate-specific receptor—hydroxycarboxylic acid receptor 1 (HCAR1)—to mediate angiogenesis, inflammation, neurogenesis, and synaptic plasticity (Shang et al., 2023; Morland et al., 2017).

Lactate serves as both an efficient energy substrate for neurons and an epigenetic signal for transcriptional regulation in the brain. Under homeostatic conditions, lactate entering neurons via the ANLS pathway is efficiently oxidized by mitochondria to generate ATP, maintaining lactylation modifications at a low basal state to ensure the prioritization of cellular energy metabolism (Allen and Lyons, 2018). Oxidative energy production primarily occurs in mitochondria and neurons with high energy demands, whereas lactylation is concentrated in the nucleus, cytoplasm, and glial cells with vigorous glycolysis (Zhang et al., 2019; Gaffney et al., 2020). However, under pathological conditions such as ischemia or inflammation, the production rate of lactate far exceeds the oxidative capacity of mitochondria. This leads to intracellular lactate concentrations surpassing the threshold for signal triggering, prompting excess lactate to flow through the Lactyl-CoA pathway toward protein lactylation modifications (Zhang et al., 2019). Consequently, lactylation can be regarded as a metabolic receptor that converts excessive metabolic stress into long-term transcriptional remodeling (Yao S. et al., 2023).

## 3 Protein Lactylation

Lactate is an important carbon-containing metabolite produced during glycolysis. Its biological roles have drawn renewed attention because of the Warburg effect in tumor cells. This has led to a deeper investigation into its regulatory mechanisms (Zhang W. et al., 2023).

Post-translational modifications (PTMs) refer to reversible chemical changes on the protein backbone or amino acid side chains. More than 650 PTM types have been identified, such as phosphorylation, ubiquitination, methylation, acetylation, glycosylation, lipidation, sulfation, succinylation, SUMOylation, crotonylation, malonylation, and redox modifications (Yang et al., 2025; Gong et al., 2024; Zhong Q. et al., 2023, Shvedunova and Akhtar, 2022). PTMs increase the chemical complexity and information capacity of proteins. They influence protein conformation, localization, stability, function, and activity, and thereby regulate many physiological and pathological processes (Lu et al., 2024; Magits et al., 2024; He et al., 2025; Ramazi and Zahiri, 2021). Maintaining PTM homeostasis is essential for human health. Abnormal PTMs can alter protein properties and functions. They may contribute to diseases such as diabetes, tumor proliferation, inflammation, and neurodegenerative disorders (Wang X. et al., 2024; Zhang X. et al., 2024; Jing et al., 2025). Lactylation (also called lysine lactylation) is a newly identified PTM driven by lactate (Zhang et al., 2019). It was first reported in 2019 and has become an important epigenetic mechanism linked to lactate function. Unlike many classical PTMs that mainly rely on enzymatic reactions, lactylation is unique because it uses lactate—a product of glycolysis—as the direct substrate for histone modification. This feature positions lactylation as a key link between cellular metabolic states and gene regulation.

### 3.1 Discovery and research progress of lactylation

With the rapid development of liquid chromatography and mass spectrometry (LC/MS), research on post-translational modifications advanced quickly, and lactylation became an important topic. In, Zhang et al. (2019) detected a mass shift of 72.021 Da on lysine residues in human breast cancer MCF-7 cells. This shift matched the mass change of a lactyl group attached to the *ε*-amino group of lysine. By conducting experiments on human lung cancer A549 cells, human cervical cancer HeLa cells, and mouse embryonic fibroblasts isotopically labeled glucose and lactate, together with immunoblotting, the team confirmed that this modification originated from endogenous or exogenous lactate. In a mouse bone marrow-derived macrophage model, researchers induced inflammatory responses with LPS and IFN-*γ*, observing elevated histone lactylation levels alongside lactate accumulation (Zhang et al., 2019). Key experimental evidence demonstrated that histone acylation modification is significantly reduced when the enzyme that converts pyruvate to lactate is knocked out, further proving its lactate dependence.

The authors also revealed the mechanism: lactate is converted to lactyl-CoA and then transferred to histone lysine residues by a lactyl-transferase. In addition, Zhang et al. (2019) identified lactylation while screening different histone acylation marks. They predicted and verified lactylation as a lactate-responsive histone modification and confirmed its presence using orthogonal validation methods. Their findings showed that lactate can modify both histone and non-histone proteins through lysine lactylation and regulate gene transcription, thereby influencing cellular functions and behavior. Other studies later confirmed that different chromatin modifications can induce distinct transcriptional states (Zhang et al., 2019). In, Gaffney et al. (2020) reported another lactylation pathway. The glycolytic by-product methylglyoxal reacts with glutathione to form lactoyl-glutathione, which is hydrolyzed by glyoxalase 2 to produce D-lactate. The lactyl group can then be transferred non-enzymatically to lysine residues. Knocking out glyoxalase 2 increased lactoyl-glutathione levels and markedly elevated lysine lactylation, demonstrating that lactoyl-glutathione is a direct substrate. An alkyne-tagged methylglyoxal analog showed enrichment of these modifications on glycolytic enzymes, suggesting a negative feedback role on glycolysis. Although these studies proposed different sources for lactylation substrates, the regulatory impact of protein lactylation on gene expression has since been widely confirmed.

When cellular metabolism undergoes reprogramming and shifts from oxidative phosphorylation to glycolysis, the structure and function of intracellular proteins adapt accordingly. Through post-translational modifications, proteins add chemical groups to amino acid residues, change their physicochemical properties, alter spatial conformation, and diversify their functions (Bradley, 2022). Lactylation has now emerged as an important new member of this regulatory system.

### 3.2 Pathways and influencing factors

**Figure 2:**
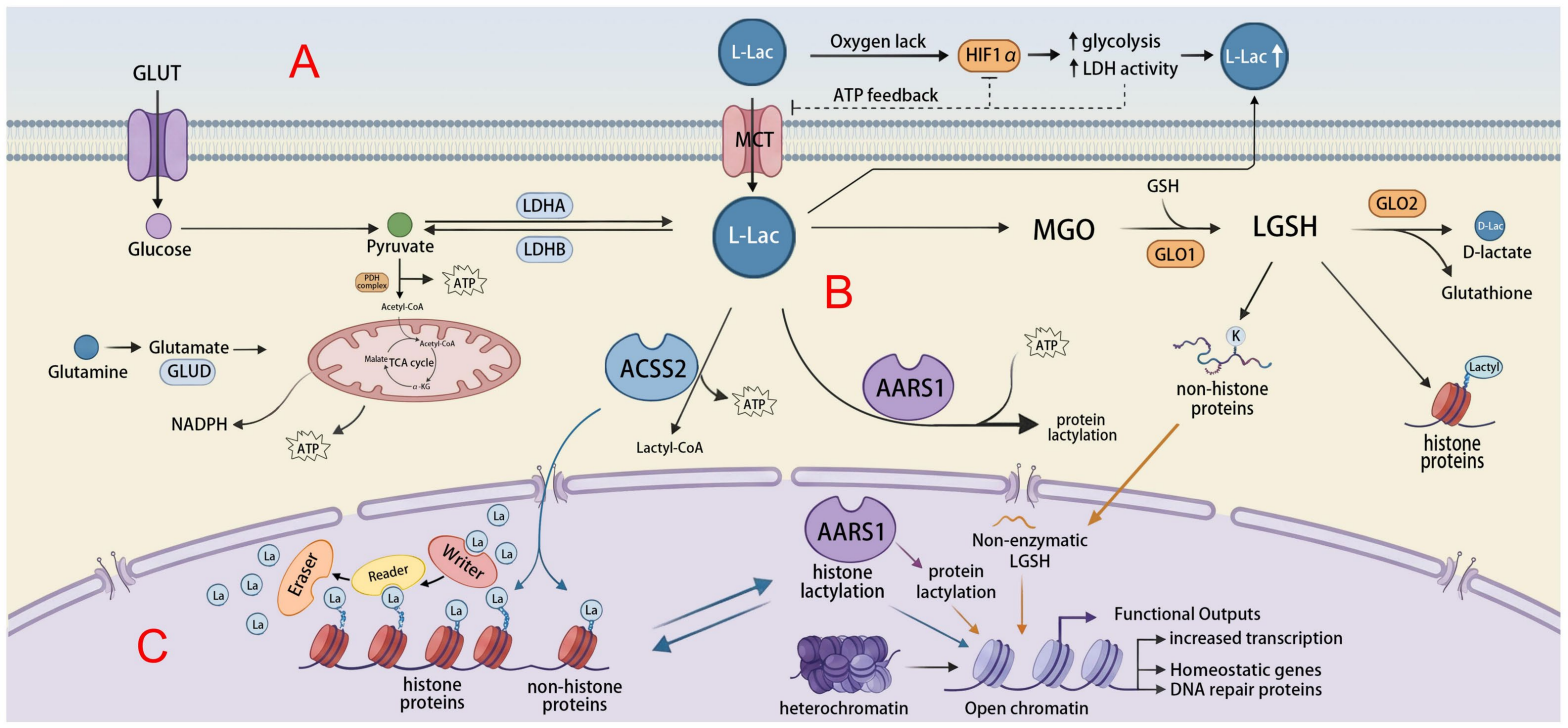
Schematic representation of cellular lactate metabolism and the mechanisms of protein lactylation. **(A)** Metabolic inputs: Glucose and glutamine metabolism fuel the TCA cycle and glycolysis. Under hypoxic conditions, HIF1α upregulation promotes glycolysis and LDH activity, leading to intracellular L-lactate accumulation and transport via MCT. **(B)** Lactylation substrate generation: Lactate is utilized for protein post-translational modification via three primary pathways: (1) The canonical synthesis of Lactyl-CoA mediated by nuclear-localized ACSS2; (2) The AARS1 enzymatic bypass, utilizing ATP to form an aminoacyl-adenylate intermediate; (3) The GLO1/2-LGSH pathway, which facilitates non-enzymatic lactylation via S-D-lactoylglutathione. **(C)** Epigenetic regulation: In the nucleus, “Writer” enzymes deposit lactyl groups onto histones, while “Erasers” remove them. This modification promotes chromatin opening (heterochromatin to euchromatin transition), driving the transcription of genes involved in homeostasis and DNA repair.

Lactylation is a post-translational modification in which a lactyl group derived from lactate is added to lysine residues. It includes three isomers: L-lactyl-lysine (KL-la), D-lactyl-lysine (KD-la), and N-*ε*-(carboxyethyl)-lysine (Kce) (Zhang D. et al., 2025). In the human body, lactate exists mainly as L-lactate and D-lactate. L-lactate is the dominant form. It is produced directly through glycolysis and accounts for more than 95% of total lactate. It also regulates lactate metabolism and signaling. D-lactate makes up only 1–5%. It comes from diet, gut bacterial metabolism, propylene glycol metabolism, or the methylglyoxal pathway (Xin et al., 2022; Monroe et al., 2019; Moreno-Yruela et al., 2022). Accordingly, two lactylation pathways exist in cells: L-lactylation (direct lactylation) and D-lactylation (indirect lactylation) (Hagihara et al., 2021). Direct lactylation uses L-lactate as the precursor. The lactyl group is supplied by lactyl-CoA (Zhang et al., 2019). This pathway was first thought to modify only histones. Later studies showed that it also occurs on many non-histone proteins, suggesting broader regulatory functions (Zhang D. et al., 2025). KL-la is the predominant lactylation form in cells and contributes to glycolysis and the Warburg effect (Zhang D. et al., 2025). D-lactylation does not use D-lactate directly. Instead, it originates from methylglyoxal (MGO), which forms S-D-lactoyl-glutathione. This intermediate donates the lactyl group through a non-enzymatic reaction (Gaffney et al., 2020; Rabbani et al., 2014). Both lactylation pathways coexist. Their relative contribution depends on protein expression in specific cell types. L-lactylation is more involved in feedback regulation of metabolic homeostasis, whereas D-lactylation may be more involved in stress response or pathological protein damage (Trujillo et al., 2024) (Figure 2).

## 4 Mechanisms of lactylation

Protein lactylation includes histone lactylation and non-histone lactylation. It can arise through enzymatic or non-enzymatic pathways. Its regulation depends on a coordinated system of “writers,” “erasers,” and “readers.” Zhang et al. (2019) proposed that lactylation occurs under the catalysis of multiple enzymes. Lactate production is linked to glycolysis and mitochondrial metabolism. Changes in intracellular lactate levels can alter the degree of lactylation. Enzymes that regulate glycolysis or mitochondrial pathways therefore influence the extent of protein lactylation. Based on its formation mechanism, lactylation can be divided into enzymatic and non-enzymatic types. The non-enzymatic pathway involves the transfer of a lactyl group from lactyl-glutathione (LGSH) to lysine residues. LGSH forms rapidly through the coupling of glyoxalase 1 with glutathione, a by-product of glycolysis. p300 acts as the acyltransferase for both histone and non-histone lactylation. Histone deacetylases (HDACs) and NAD-dependent deacetylases (sirtuins, SIRT1–7) function as deacetylases. Histone lactylation is an epigenetic modification that activates gene transcription. It alters chromatin structure, modulates transcriptional activity, and regulates gene expression (Zhang et al., 2019). In pancreatic ductal adenocarcinoma, histone lactylation activates TTK protein kinase and BUB1B transcription, which drives tumor progression (Li F. et al., 2024). Non-histone lactylation can modify protein stability, activity, and function. For example, lactylation of hypoxia-inducible factor-1α (HIF-1α) increases its stability under normoxia and promotes angiogenesis in prostate cancer (Luo et al., 2022). Through these mechanisms, lactate regulates gene expression and protein behavior, ultimately shaping various biological processes and disease pathways. Lactylation is associated with a broad range of biological functions. These include macrophage polarization (Chen et al., 2022), chromatin remodeling (Dai et al., 2022), and cell differentiation (Zhou et al., 2024). Later studies further demonstrated its roles in cancer (Li W. et al., 2024), heart failure (Uyar et al., 2020), autoimmune diseases (Huang et al., 2024), neuronal excitation (Hagihara et al., 2021), inflammation (Wang N. et al., 2022), and neurodegeneration (Schirinzi et al., 2020).

### 4.1 Enzymatic lysine lactylation

Histone lactylation is a lysine post-translational modification that occurs in the nucleus. In this process, lactate is transferred to lysine residues under the catalysis of acetyltransferase enzymes, producing a lactyl group. This reaction alters histone structure and chromatin activity (Zhang D. et al., 2025). Intracellular lactate can be converted to lactyl-CoA, which serves as the direct high-energy donor for lysine lactylation (Kla). Early studies noted that the specific enzyme responsible for activating lactate to lactyl-CoA in mammals had not been clearly identified. However, the presence of lactyl-CoA can be quantified using subcellular fractionation combined with targeted LC-HRMS or LC–MS/MS techniques (Zhang D. et al., 2025; Varner et al., 2020).

Recent studies offer more direct evidence. First, histone lactylation assays show that lactyl-CoA can be used as a co-substrate by several KAT enzymes, including p300, HBO1, and KAT2A. Second, new enzymatic and cell-based studies report that ACSS2 can translocate into the nucleus under specific stimuli. It then catalyzes the conversion of L-lactate into lactyl-CoA and cooperates with enzymes like KAT2A to regulate histone lactylation and downstream gene expression. This mechanism contributes to pathways such as tumor immunity (Zhu et al., 2025). In addition, non-canonical “lactyl-transfer” routes may exist. For example, AARS1 can catalyze protein lactylation through a lactate-AMP intermediate (Zong et al., 2024). This suggests that the enzymatic and auxiliary pathways of Kla may coexist. Overall, evidence supports lactyl-CoA as the direct donor for Kla. Enzymes such as ACSS2 can generate lactyl-CoA locally within the nucleus, but the dominant lactyl-CoA–producing enzymes likely vary across cell types and under different physiological or pathological states, and require further investigation (Zhang D. et al., 2025). Functional studies show that lactate induces the expression of profibrotic factors in macrophages. The accompanying increase in histone lactylation can worsen pulmonary fibrosis (Cui et al., 2021). In ocular melanoma, elevated histone lactylation can silence several tumor suppressor genes and accelerate tumor development. Suppressing aberrant histone lactylation significantly slows melanoma progression (Yu J. et al., 2021).

Lactyl-CoA stimulates histone lactylation on specific DNA regions. This enzymatic process mainly involves three classes of enzymes: writers, erasers, and readers (Gao et al., 2024). The lactylation “writers” attach lactyl groups to defined protein sites. They catalyze the esterification of endogenous or exogenous L-lactate with lactyl-CoA and transfer the lactyl group to the *ε*-amino group of lysine residues on histone and non-histone proteins. This modification alters the conformation and biological functions of target proteins. Lactylation “readers” recognize the modified lysine residues with high specificity. Their binding triggers downstream signaling cascades and mediates the corresponding biological effects. When signal transduction is completed, lactylation “erasers” remove the modification. They eliminate the lactyl group from lysine residues and terminate lysine lactylation on the target proteins (Zhao et al., 2025; Sabari et al., 2017).

#### 4.1.1 Writers

Current studies identify histone acetyltransferases as the major “writers” of lactylation. Among them, p300 (E1A binding protein p300) is the most extensively investigated (Zeng et al., 2023; Xu et al., 2023). Additional enzymes have also been reported as novel lactyltransferases, including alanyl-tRNA synthetase (AARS1/2) (Zong et al., 2024; Li H. et al., 2024; Ju et al., 2024), the acetyltransferase Tip60 (Chen et al., 2024), YiaC (a prokaryotic lactyltransferase) (Dong et al., 2022), and lysine acetyltransferase 8 (KAT8) (Xie et al., 2024). In the CNS, p300 participates in the physiological processes of various diseases by mediating histone and non-histone lactylation. Pre-treatment with a small-molecule p300 inhibitor markedly reduced lactylation levels in a murine model of ischemic stroke, thereby preserving neuronal activity and mitigating ischemic brain injury (Xiong et al., 2024). In AD, p300, as a key “writers” of tau protein lactylation, mediates the elevation of histone H4K12la modification levels, inhibits the ubiquitination degradation of Tau protein, and exacerbates its pathological accumulation (Zhang X. et al., 2025). Recent breakthrough findings have identified GTPSCS as lactyl-CoA synthase in glioma cell nuclei, which promotes glioma progression through p300-mediated histone lactylation (Liu et al., 2025).

#### 4.1.2 Erasers

Class I and class III histone deacetylases (HDAC1–3) and the sirtuin proteins SIRT1–3 are the typical “erasers” that remove lactyl groups. Accumulating *in vitro* evidence indicates that nuclear HDAC1–3 are the most potent lysine delactylases, whereas SIRT1–3 also exhibit substantial delactylation activity toward both L- and D-lactyl modifications. In the CNS, SIRT2 is the most abundant longevity protein (Sirtuins), particularly in oligodendrocytes and neuronal somata. Compared to the systemically expressed HDAC1/3, SIRT2 exhibits a more specific response to metabolic stress in the brain, potentially serving as a core deacetylase for regulating non-histone acetylation in the brain (Zu et al., 2022). Jennings et al. (2021) using chemical biology approaches and CRISPR-Cas9 screening, identified the NAD^+^-dependent deacetylase SIRT2 as an efficient remover of non-enzymatic acyl modifications. It is the first recognized eraser for non-enzymatic lysine lactylation. Zu et al. (2022) used fluorescent probes containing lactyl-lysine residues and confirmed in glial cells that SIRT1, SIRT2, SIRT3, and SIRT5 can catalyze delactylation. Among them, SIRT2 shows the strongest activity, and this activity is inhibited by Tenovin-6. SIRT1 likely serves as a deacetylase for non-histone proteins, while HDAC1–3 are the major histone deacetylases in cells. These enzymes remove multiple PTMs and are essential for neuronal development and differentiation (Tang et al., 2019). Inhibition of HDAC1-3 has been demonstrated to regulate neuronal identity and activate transcription during mouse neurodevelopment. The chromatin modifications of H3K9, H3K9 acetylation, and H3K18 lysine acetylation dynamically change during embryonic development, interacting with other PTMs to determine chromatin accessibility and neuronal fate (Dai et al., 2022).

#### 4.1.3 Readers

Protein lactylation appears to be reversible under the regulation of lactylation and delactylation enzymes. However, it remains unclear which proteins function as readers in this process and how they contribute to downstream regulation. At present, no widely accepted lactylation “readers” have been identified. The lactylation labeling is recognized by specific reader proteins, which play a critical role in gene expression regulation during macrophage polarization, neuronal synaptic plasticity, and stress responses by altering chromatin accessibility or recruiting transcription factors (Hagihara et al., 2021). Recent studies have identified bromodomain-containing proteins as potential readers of lactylation, capable of recognizing lactoylation modifications and regulating the transcriptional activity of target genes. For instance, in the subarachnoid hemorrhage model, bromodomain-containing protein 4 (BRD4) was found to bind to H4K8la in astrocytes, and its expression silencing significantly affected the level of this modification, suggesting that BRD4 may serve as a potential reader of H4K8la under this pathological context (Zhang F. et al., 2024). Transcription factor Dux promotes lactate accumulation and drives histone lactylation modifications (e.g., H3K18la) by activating glycolytic metabolism. These modifications are subsequently specifically recognized by chromatin remodeling factor Brg1, which activates downstream genes by opening chromatin structure, thereby advancing the process of cellular reprogramming (Hu et al., 2024). This mechanism is not only critical in somatic cell reprogramming but also provides a novel perspective on the metabolic-epigenetic coupling in neurodevelopment and plasticity. These findings collectively reveal the potential pivotal role of the “write-erase-read” dynamic network—from lactate metabolism to epigenetic regulation—in neural function and disease. Their roles remain preliminary, and they represent promising targets for further investigation (Table 1).

**Table 1:** Functions and characteristics of lactylation-regulating enzymes.

Regulatory molecule type	Specific molecule	Core features	References
Writers	P300	The primary acetyltransferase involved in lactylation mediates the lactylation of histones and non-histone proteins.	Zeng et al. (2023), Xu et al. (2023), Dong et al. (2022), Zhang N. et al. (2023), Zhang X. et al. (2025)
	AARS	As a new type of lactate transferase, it participates in the process of lactyl transfer	Zong et al. (2024), Li H. et al. (2024), Ju et al. (2024)
	Tip60	Mediation of lactyl transfer reaction in lactylation modification	Chen et al. (2024)
	YiaC	Lactate synthase is involved in the regulation of the lactylation modification of metabolic enzymes by binding to lactyl-CoA	Dong et al. (2022)
	KAT8	The process of lactyl transfer in the modification of lactylation	Xie et al. (2024)
Erasers	HDAC 1–3	1. The main histone deacetylase in cells; 2. Efficiently removes lysine lactylation modification, which is crucial for neuronal development and differentiation	Moreno-Yruela et al. (2022), Tang et al. (2019)
	SIRT1-3	NAD+-dependent deacetylases that catalyze the removal of lactyl groups. SIRT2 exhibits the highest physiological activity among them and can be specifically inhibited by Tenovin-6.	Moreno-Yruela et al. (2022), Zu et al. (2022)
	SIRT5	The catalytic activity of acyl group removal and the reverse regulation of lactylation modification	Zu et al. (2022)
Readers	Brg1	Chromatin remodeling factor that specifically recognizes lactylation modifications (e.g., H3K18la) induced by Dux, activates downstream genes by opening chromatin structure, and advances cellular reprogramming; critical for metabolic-epigenetic coupling in neurodevelopment and plasticity.	Hu et al. (2024)
	BRD4	Bromodomain-containing protein; binds to H4K8la in astrocytes in a subarachnoid hemorrhage model; its silencing significantly affects H4K8la levels, suggesting it acts as a potential reader under this pathological context.	Zhang F. et al. (2024)

### 4.2 Non-enzymatic lysine lactylation

Indirect lactylation uses methylglyoxal as a precursor. In tissues with high glycolytic activity, methylglyoxal reacts with glutathione to form S-D-lactoylglutathione, which donates the lactyl group for non-enzymatic lactylation of non-histone proteins. Because this process does not require enzymatic catalysis, it is described as “non-enzymatic lysine lactylation.” This modification has been identified in hepatocellular carcinoma, where it promotes tumor proliferation and metastasis (Gaffney et al., 2020; Yang et al., 2023). In human embryonic kidney cells, more than 350 proteins carry non-enzymatic lysine lactylation. The modification level depends on the intracellular concentration of lactoylglutathione, which rises markedly when glycolysis is reduced (Gaffney et al., 2020). Non-histone lysine lactylation is a newly recognized PTM that involves the addition of a lactyl group to lysine residues. Gaffney et al. (2020) showed that the acyl donor originates from lactoylglutathione, a glycolytic intermediate. Lactoylglutathione can be directly transferred to protein lysine residues to produce the lactylated form. In cells, lysine lactylation correlates with lactoylglutathione abundance. Glyoxalase 2 (GLO2) is the only known enzyme capable of hydrolyzing lactoylglutathione, making it a key regulator of both lactoylglutathione levels and non-enzymatic lactylation. Zhang D. et al. (2025) demonstrated that impairing the glyoxalase pathway selectively disrupts the formation of Kce and Kd-la, whereas the production of lactoyl-CoA is associated with increased Kl-la. L-lactate also inhibits lactoylglutathione degradation, raising its intracellular level and thereby promoting D-lactylation (Hagihara et al., 2021). In mouse neurons, proteins modified through the indirect pathway have been detected (Hagihara et al., 2021). These findings suggest that direct and indirect lactylation coexist rather than exclude each other, and the dominant pathway likely depends on protein expression patterns in brain cells (Hagihara et al., 2021; Gaffney et al., 2020).

## 5 Sites of protein lactylation

Protein lactylation includes histone lactylation and non-histone lactylation. Evidence from multiple techniques and sample types has confirmed these modifications. Early evidence of histone lactylation came from high-performance liquid chromatography–tandem mass spectrometry. In that study, lactylation was detected on lysine residues. The mass shift matched the addition of a lactyl group to the *ε*-amino group of lysine. Lactylation at this stage was observed on core histone H3 (Zhang et al., 2019). Zhang et al. (2019) further used mass spectrometry to identify several histone lactylation sites in human MCF-7 cells and mouse bone-marrow–derived macrophages. They also confirmed that the H3K18 site in M1 macrophages undergoes lactylation. Lactylated H3K18 directly regulates the transcription of arginase 1 (Arg1). This type of lactylation on core histones can induce chromatin gene transcription and promote the expression of homeostatic genes such as Arg1. These changes jointly drive macrophage polarization from the M1 to the M2 phenotype (Ma et al., 2022). A social defeat stress model identified 63 candidate lysine-lactylated proteins in the mouse prefrontal cortex. Stress preferentially increased lactylation on linker histone H1. This finding shows that lactylation is common in neuronal cells. Neuronal excitation can further enhance H1 lactylation (Hagihara et al., 2021). Lactylation is also involved in embryonic development. In sheep endometrium, the lactylation level of histone H3 at lysine 18 (H3K18la) is relatively high. H3K18la helps remodel uterine receptivity and ensures successful embryo implantation (Yang Q. et al., 2022). Yang et al. (2021) used immunofluorescence with specific antibodies and found lactylation on pan-histone, H3K23, and H3K18 in oocytes and pre-implantation embryos. They also observed that hypoxia reduced LDHA expression, lowered lactate production, and decreased lactylation levels. Reduced lactylation may impair embryonic development and disrupt embryonic gene expression. Both endogenous and exogenous lactate increase histone lactylation (Li et al., 2020). However, interactions among different lactylation sites create a more complex outcome. When lactate levels rise, H3K18la and H3K24la increase in mouse embryonic stem cells, but H4K8la and H4K18la decrease. This inconsistency may arise from specific transcription factors or coactivators (e.g., p300/CBP) activating certain feedback mechanisms when lactate concentration increases, or from the higher activity of de-lactylating enzymes (e.g., HDACs) at specific loci, leading to differential expression of elevated H3 and reduced H4 at certain sites (Tian and Zhou, 2022).

For non-histone lactylation, a global lysine lactylome analysis of Botrytis cinerea was performed using liquid chromatography–tandem mass spectrometry. The study identified 273 lactylated lysine residues on 166 proteins. Forty-three ribosomal structural proteins carried lactylation marks. Most lactylated proteins were widely distributed in the nucleus (36%), mitochondria (27%), and cytoplasm (25%) (Gao et al., 2020). In *Trypanosoma brucei*, 387 lactylation sites were detected on 257 proteins. Heat shock protein 90 (HSP90) contained 14 lactylated lysine residues (Zhang N. et al., 2021). Wan et al. (2022) showed that ammonium adducts can improve the identification of lactylated sites. Using this method, they detected 125 sites on 83 proteins from 14 human cell lines in the Meltome Atlas. They also found extensive lactylation on glycolytic enzymes. Lactylation at lysine 147 of fructose-bisphosphate aldolase A (ALDOA) inhibited ALDOA activity through negative feedback and reduced glycolytic flux. Yang K. et al. (2022) reported that the non-histone protein HMGB1 also undergoes lactylation. Beyond traditional mass spectrometry, researchers designed a sodium L-lactate derivative, (S)-2-hydroxypent-4-ynoate (YnLac). YnLac can directly target lysine residues and enables the detection of lactylated proteins in mammalian cells. Using this tool, many new non-histone lactylation sites were identified, which expanded the known substrate scope of protein lactylation (Sun et al., 2022). These findings show that lactylation is not limited to histones. It also occurs on nuclear proteins, organelle proteins, and cytosolic proteins, suggesting a broad regulatory role in cellular processes (Yang D. et al., 2022). In addition, a study on non-enzymatic lactylation identified 350 lactylated proteins. DAVID and KEGG analyses revealed that these proteins were mainly enriched in glycolysis and carbon metabolism pathways (Gaffney et al., 2020). This further indicates that lactylation may regulate biological activities through multiple pathways and plays an important role in cellular function (Table 2.)

**Table 2:** Summary of histone and non-histone lactylation targets and their regulatory mechanisms.

Modification type	Modifying site/protein	Sample type	Regulatory mechanism	References
Histone lactylation	H3K18	Human MCF-7 cells and mouse bone marrow derived macrophages	Direct induction of chromatin gene transcription regulates the expression of homeostasis genes such as Arg1	Zhang et al. (2019), Ma et al. (2022)
	H3K18la	Sheep endometrium	Reconstructing uterine receptivity to create conditions for embryo implantation	Yang Q. et al. (2022)
	H3K18, H3K23	Oocyte, preimplantation embryo	Regulation of Embryonic Gene Expression by Hypoxia (Hypoxia Reduces lactylation Level)	Yang et al. (2021)
	H1	Prefrontal cortex of mouse	Response to stress signals and neural excitation, lactate modification occurs first	Hagihara et al. (2021)
	H3K18la, H4K8la	Mouse embryonic stem cells (mESCs)	Lactate can improve the modification level of the protein under the regulation of endogenous and exogenous lactate	Zhang et al. (2019), Li et al., (2020), Tian and Zhou (2022)
Non-histone lactylation	273 lysine residues of 166 proteins (including 43 ribosomal structural proteins)	Fungal species: *Aspergillus griseus*	Widely distributed in the nucleus, mitochondria, and cytoplasm, it participates in various intracellular processes.	Gao et al. (2020)
	387 sites of 257 proteins (HSP90 contains 14 lysine residues)	*Trypanosoma brucei*	Multiple sites of lactylation modify and expand the diversity of non-histone lactylation substrates	Zhang N. et al. (2021)
	ALDOA K147	14 human cell lines	ALDOA K147 Lactylation Inhibits Its Activity Through Negative Feedback Regulation and Participates in the Regulation of Glycolytic Enzyme Modification	Wan et al. (2022)
	HMGB1	Macrophage	Macrophages uptake extracellular lactate via MCT, promoting HMGB1 lactylation and confirming its lactylation modification.	Yang K. et al. (2022)
	Multiple new non-histone sites in mammalian cells	Mammalian cell	Identification of novel modification sites via YnLac-targeted binding to protein lysine residues	Sun et al. (2022)
	350 kinds of lactate modified proteins		Modifying proteins are enriched in glycolysis and carbon metabolism pathways and participate in pathway regulation	Gaffney et al. (2020)

## 6 Detection methods for protein lactylation

In recent years, advances in bioinformatics have greatly facilitated the investigation of biological processes. Mass spectrometry and liquid chromatography–mass spectrometry can identify protein lactylation sites, but these approaches are time-consuming and labor-intensive. To improve the efficiency of lactylation site prediction, researchers have developed a series of machine-learning–based computational tools. These tools have become an important supplement to traditional experimental detection. By training on sequence and structural features of known lactylation sites, machine-learning models can rapidly detect potential modification patterns. They significantly enhance large-scale screening and reduce both experimental time and cost. Examples include the few-shot learning–based prediction server^1^ (Jiang et al., 2021), the DeepKla platform integrating neural networks^2^ (Meng et al., 2022), and the Auto-Kla tool based on automated machine learning^3^ (Lai and Gao, 2023). The development of these tools marks a shift from an experiment-driven approach to a combined “experiment + computation” framework. This transition improves research efficiency and promotes more practical and reliable computational prediction of lactylation sites. For instance, when Yao et al. sought to investigate the pivotal role of lactylation in cerebral ischemia–reperfusion injury (CIRI), they first identified upregulated and downregulated sites of lactylation across various proteins through proteomics. Subsequently, they explored the biological pathways of differentially expressed proteins in the database, ultimately elucidating the potential impact of lactylation modifications (Yao Y. et al., 2023).

It is noteworthy that over-reliance on machine learning predictions without adequate LC–MS validation may mislead the understanding of brain lactation modification profiles. Artificial intelligence approaches are advancing the accuracy and efficiency of protein post-translational modification recognition, with computational models enabling accelerated site screening, identification of sequence features, and providing a theoretical foundation for precision medicine. However, rigorous experimental validation remains the cornerstone for confirming prediction reliability and functional relevance. Integrated development holds promise for deepening the understanding of lactation’s role in neurological disorders and guiding the design of rational therapeutic interventions based on pathological mechanisms.

## 7 Protein lactylation and brain disorders

The brain consists of distinct cells with unique metabolic programs. This inherent metabolic heterogeneity determines fundamental differences in how neurons, astrocytes, and microglia perceive, process, and respond to lactate accumulation. Consequently, the lactate-lactation axis serves as a cell-type-specific metabolic interpreter, transforming the same extracellular lactate signal into different functional outputs within neural networks.

Astrocytes, as the primary lactate producers via aerobic glycolysis, maintain a high glycolytic flux (Zhou et al., 2024; Dias et al., 2023). They express high levels of the lactate exporter MCT4 (Ma et al., 2024), creating a steep outward lactate gradient. While constantly exposed to self-generated lactate, their intracellular lactylation landscape may be tuned for homeostatic feedback regulation, perhaps modifying glycolytic enzymes or proteins involved in the astrocyte-neuron lactate shuttle to maintain metabolic coupling (Allen and Lyons, 2018). Under pathological stress, this system may be hijacked, leading to lactylation of pro-inflammatory or fibrotic transcripts (Zhou et al., 2024).

Neurons are lactate consumers with a high oxidative capacity and reliance on mitochondrial ATP (Dias et al., 2023). They express the high-affinity lactate importer MCT2 (Arnsten et al., 2012). For neurons, lactate is primarily an alternative fuel. However, when oxidative metabolism is impaired (e.g., in ischemia or neurodegeneration) or when lactate influx exceeds metabolic capacity, the resulting rise in intracellular lactate may trigger a lactylation program geared toward stress response and survival decisions. Lactylation in neurons might preferentially target proteins involved in synaptic plasticity, excitability, autophagy, or apoptosis, differentiating its functional impact from that in glia (Dias et al., 2023).

Microglia are lactate-sensitive metabolic plasticity. In a surveillant state, they rely on oxidative phosphorylation. Activated microglia rapidly switch to aerobic glycolysis (Han et al., 2025). This activation-coupled lactate surge likely drives a dynamic lactylation program that is integral to their phenotypic polarization. Enhanced lactate sensitivity, moderate lactate accumulation can induce histone lactylation, thereby regulating LPS-stimulated microglia and M1/M2 polarization in AD/PD mouse models (Li et al., 2022; Lian et al., 2024; Abdolmaleky et al., 2019; Bubber et al., 2005).

Various epigenetic modifications and their interacting molecules work together to regulate gene-specific expression. This process establishes the chromatin state across different genomic regions. Dysregulated metabolic activity and epigenetic alterations are often associated with many diseases (Griazeva et al., 2023). Abnormal epigenetic mechanisms can drive disease development and also reveal potential therapeutic targets. Therefore, the establishment and change of cellular functional states during physiological and pathological processes are shaped by epigenetic regulation. This makes epigenetics essential for understanding both health maintenance and disease progression (Agbleke et al., 2020). Histone PTMs also influence many aspects of neuronal development and function, highlighting the importance of epigenetic mechanisms in brain health (Prasanth et al., 2024).

**Figure 3:**
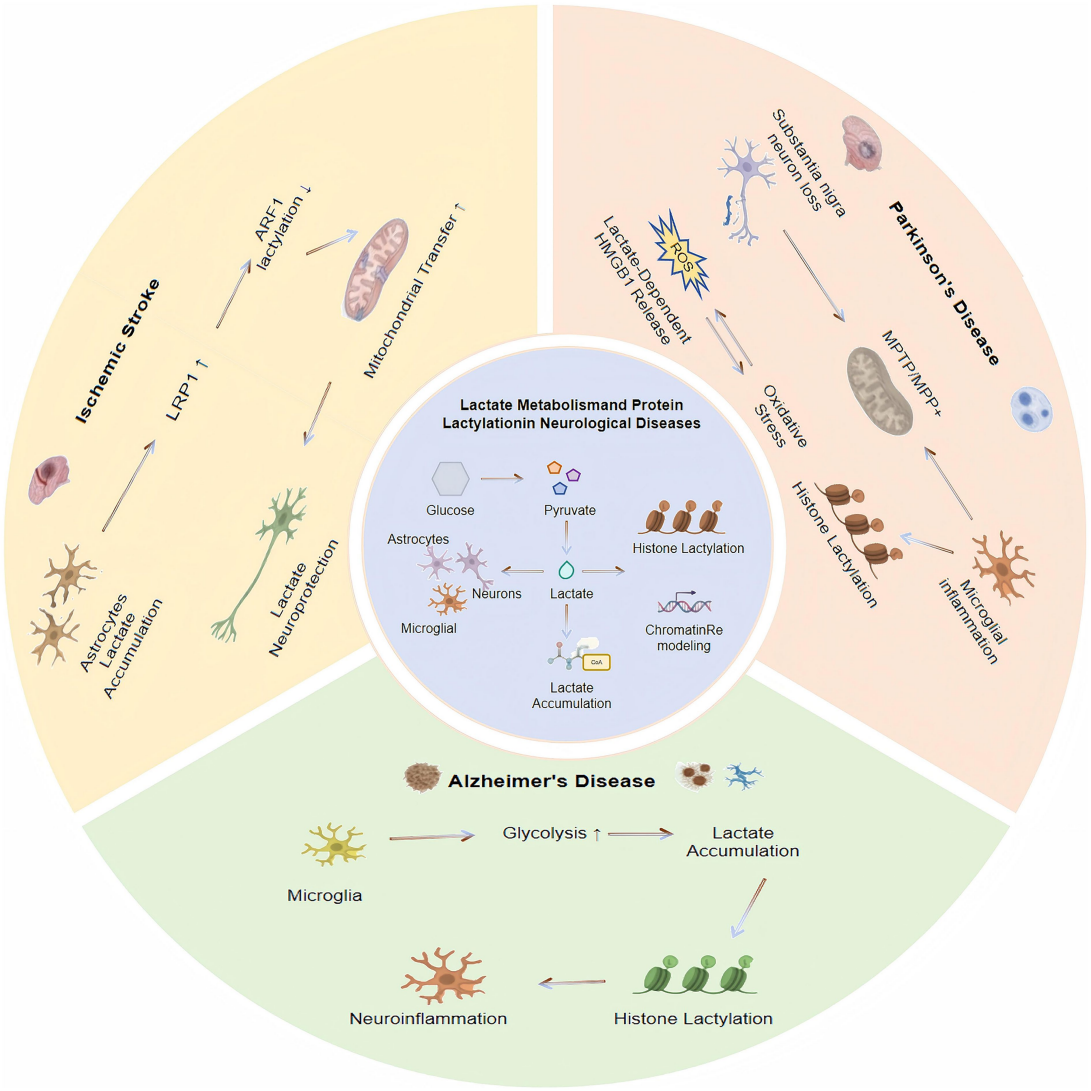
Regulatory roles of lactate metabolism and lactylation in brain disorders. Schematic illustration summarizing how dysregulated lactate metabolism and protein lactylation contribute to the pathogenesis of ischemic stroke, neurodegenerative diseases, epilepsy, psychiatric disorders, and glioma through metabolic, inflammatory, and epigenetic mechanisms.

Lactate has several beneficial effects on the brain. It can improve memory decline, enhance cerebral blood flow, optimize brain energy metabolism, alleviate neurological deficits, and promote neural regeneration (Hu et al., 2021; Lambertus et al., 2021). Emerging data show that lactate regulates transcriptional activity, cellular functions, and disease processes. Many central nervous system (CNS) disorders feature neuron loss, atrophy in specific brain regions, and disruption of neural networks due to progressive neuronal death (Abdolmaleky et al., 2019). Neuroinflammation, a CNS inflammatory response, is driven by cytokines and chemokines released from microglia, astrocytes, endothelial cells, and peripherally derived immune cells. Acute neuroinflammation plays a protective role against toxins, injury, and infection. As innate immune cells, microglia are essential for responding to damage, driving neurodegenerative disease progression, and maintaining CNS homeostasis (Woodburn et al., 2021). Microglial dysfunction disrupts normal brain physiology and can trigger disease. This impairment is linked to mitochondrial dysfunction and lactate accumulation (Hagihara et al., 2018). Elevated lactate promotes a shift in microglial polarization through lysine lactylation and acts as an internal “accelerator” of the microglial lactate timing mechanism (Han et al., 2023). In addition, increased histone lactylation may influence the pathophysiology of brain disorders (Chen et al., 2022) (Figure 3).

### 7.1 Ischemic stroke

Cerebral infarction disrupts cerebral blood flow, causing local ischemia, hypoxia, and neuronal injury, and remains a leading cause of adult disability and death (Lekoubou et al., 2023). Ischemic stroke progresses through two phases: ischemia and reperfusion, during which lactate exerts distinct effects. During ischemia, astrocytes produce and accumulate lactate, which elevates intracellular protein lactylation and exacerbates neuronal injury. Suppressing lactate production at this stage reduces lactylation, limits neuronal death, and mitigates tissue damage (Xiong et al., 2024). After reperfusion, exogenous lactate confers neuroprotection independent of lactylation, while rapid glucose supply may overwhelm oxidative metabolism, enhancing glycolysis and causing abnormal lactate accumulation (Magistretti and Allaman, 2018; Xiong et al., 2024; Berthet et al., 2009). Exogenous lactate can enter mitochondria as an oxidizable substrate for metabolic processes, exerting neuroprotective effects by improving energy supply, and its effects are independent of lactation modification. In contrast, rapid and excessive glucose supplementation may lead to metabolic overload, enhance glycolysis, and result in abnormal lactate reaccumulation, exacerbating metabolic disturbances (Xiong et al., 2024). Therefore, lactate exhibits stage-specific effects during different phases of stroke: the ischemic phase is primarily characterized by lactation-mediated pathological effects, while the reperfusion phase is more functionally associated with metabolic substrates. Early restoration of blood flow and timely endovascular therapy remain essential to reduce mortality and complications (Magistretti and Allaman, 2018; Xiong et al., 2024).

Mechanistically, ischemia–reperfusion injury involves excitotoxicity, energy failure, oxidative stress, calcium overload, mitochondrial dysfunction, inflammation, acidosis, and blood–brain barrier disruption (Weinstein et al., 2004; Berthet et al., 2009; Kalogeris et al., 2012; Zhao et al., 2022). Lactylation contributes to these processes: in the rat cerebral ischemia models, lymphocyte cytosolic protein 1 (LCP1) lactylation increases significantly, and inhibiting glycolysis reduces LCP1 lactylation, promotes its degradation, and alleviates infarct progression (Haas et al., 2016; Zhang W. et al., 2023). Astrocyte-derived lactylation further aggravates neuronal death and glial activation under ischemic and hypoxic conditions (Xiong et al., 2024). Moderate lactate supplementation after ischemia–reperfusion improves neurological outcomes more effectively than glucose (Berthet et al., 2009).

At the molecular level, astrocytes are recognized as crucial neurotrophic supporters and glycogen reservoirs for neurons, playing a pivotal role in maintaining neuronal health (Guo et al., 2021). LRP1 in astrocytes decreases lactate production and suppresses lactylation of ADP-ribosylation factor 1, facilitating mitochondrial transfer to neurons and attenuating injury (Zhou et al., 2024). LRP1 exerts significant effects on neuronal function and early development by modulating synaptic generation and neuronal activity. In cerebral ischemia–reperfusion injury, LRP1 in astrocytes promotes mitochondrial transfer from astrocytes to neurons by reducing lactate production and ADP-ribosyltransferase 1 lactylation, thereby mitigating damage. ARF1 is a cytoplasmic protein that facilitates vesicular transport. LRP1 enhances the release and transfer of healthy mitochondria to neurons by inhibiting glycolysis, lactate production, and ARF1 lactylation in astrocytes, consequently alleviating ischemia–reperfusion injury in a mouse model of ischemic stroke (Zhou et al., 2024). Lactylation of Ca^2+^-signaling proteins contributes to calcium overload, mitochondrial dysfunction, and neuronal apoptosis (Yao Y. et al., 2023). Yao Y. et al. (2023) analyzed cortical protein lactylation in rats with cerebral ischemia–reperfusion injury, identifying VDAC1 and mitochondrial carriers SLC25A4 and SLC25A5 as key targets. In CIRI, SLC25A4 and SLC25A5 lactylation increased, whereas VDAC1 lactylation decreased, linking lactylation to Ca^2+^ dysregulation, mitochondrial impairment, and neuronal death. Unlike other CNS disorders, cerebral ischemia’s lactylation dysregulation is uniquely driven by acute hypoxia-induced mitochondrial dysfunction and ANLS collapse—key pathogenic targets include LCP1 and ARF1 lactylation, which specifically mediate calcium overload and mitochondrial transfer impairment. This acute metabolic crisis-driven lactylation is distinct from the chronic, inflammation-linked lactylation in AD and PD.

Taken together, lactylation exerts a phase-dependent role in ischemia–reperfusion injury: detrimental during ischemia by promoting calcium overload and mitochondrial dysfunction, yet potentially protective after reperfusion when lactate acts independently of lactylation. Temporal modulation of lactate metabolism and protein lactylation may therefore represent a promising therapeutic strategy in ischemic stroke.

### 7.2 Alzheimer’s disease

Alzheimer’s disease (AD) is a prevalent neurodegenerative disorder characterized by *β*-amyloid (Aβ) plaque deposition, tau aggregation, neuroinflammation, and age-related neuronal loss (Ramirez et al., 2025, Scheltens et al., 2021). Microglial dysregulation and metabolic reprogramming play central roles in AD pathogenesis (Nadeem et al., 2025; Calsolaro and Edison, 2016; Pan et al., 2022; Wei et al., 2023). Under normal conditions, microglia maintain neuronal function through synaptic pruning, but in early AD, hyperactive neurons trigger aberrant microglial phagocytosis, causing synaptic loss and disrupted neural signaling (Zhong L. et al., 2023). Activated microglia release pro-inflammatory cytokines and chemokines, with lactate enhancing this inflammatory response (Calsolaro and Edison, 2016). Dysregulated neuroimmune signaling, including the classical complement cascade, further contributes to synaptic dysfunction, and blocking this pathway can protect synapses and memory, highlighting the microglia–synapse axis as a potential therapeutic target (Zhong L. et al., 2023, Badimon et al., 2020; Anwar and Rivest, 2020). Studies have indicated that elevated H4K12la levels in microglia near Aβ enhance glycolytic gene transcription and exacerbate microglial dysfunction in AD (Zhang et al., 2019). In the early stages of AD, the brain often exhibits abnormal enhancement of glycolysis, leading to elevated lactate levels. High concentrations of lactate, under the action of specific enzymes, can covalently link to lysine residues of the tau protein as a post-translational modification molecule, forming lactation. This modification directly alters the conformation and function of the tau protein, reducing its ability to bind to microtubules and causing it to dissociate from them. The dissociated modified tau protein becomes more sensitive to phosphorylation, making it more prone to hyperphosphorylation, misfolding, and abnormal aggregation (Zhang X. et al., 2025). This process gradually leads to the formation of oligomers and insoluble fibrils within the cytoplasm, ultimately accelerating the development of neurofibrillary tangles, a core pathological feature. Additionally, activated microglia and other immune cells can further influence the modification status of tau in adjacent neurons through paracrine lactate. Therefore, lactation serves as a key molecular bridge connecting brain metabolic disorders with tau protein pathology.

Lactate metabolism and histone lactylation are key modulators of AD progression. Elevated histone lactylation is observed in AD patient brains and mouse models (Pan et al., 2022). In microglia, a glycolysis–H4K12la–PKM2 feedback loop drives overactivation, whereas PKM2 inhibition ameliorates pathology and improves cognition. Impaired TCA cycle activity and reduced IDH3β exacerbate lactate accumulation, which increases lactylation and induces PAX6 expression, forming a positive feedback loop that promotes tau hyperphosphorylation, Aβ accumulation, and synaptic dysfunction (Abdolmaleky et al., 2019; Bubber et al., 2005). Restoring IDH3β and reducing PAX6 normalizes metabolism, lowers histone lactylation, and mitigates AD pathology (Wang X. et al., 2024).

Cellular senescence further contributes to lactate dysregulation in microglia (Gao et al., 2020; Calsolaro and Edison, 2016; Rim et al., 2024). Senescent microglia shift toward aerobic glycolysis, elevating H3K18la in the hippocampus of aged and AD mice. H3K18la activates NF-κB signaling and increases SASP factors such as IL-6 and IL-8, accelerating brain aging and AD progression (Wei et al., 2023). In AD models, H4K12la and H3K18la differentially regulate glycolytic and inflammatory pathways, respectively, highlighting the role of lactate-driven metabolic and epigenetic reprogramming in microglial dysfunction (Nohesara et al., 2024). AD-specific lactylation dysregulation centers on the glycolysis-H4K12la-PKM2 positive feedback loop in hippocampal microglia and tau lactylation in vulnerable neurons—this dual-cell lactylation network directly promotes Aβ phagocytosis impairment and tau pathological aggregation (Zhao and Xu, 2022), a mechanism not observed in ischemia-induced acute injury or PD’s dopaminergic neuron-specific degeneration.

Taken together, lactate-induced histone lactylation integrates metabolic and inflammatory signals in microglia, exacerbating synaptic loss and neurodegeneration in AD. Therapeutically, targeting lactate metabolism or reversing aberrant lactylation in microglia may offer a promising strategy to slow disease progression.

### 7.3 Parkinson’s disease

Parkinson’s disease (PD) is a prevalent neurodegenerative disorder characterized primarily by progressive loss of dopaminergic neurons in the substantia nigra and subsequent striatal dopamine depletion (Morris et al., 2024). Lewy bodies, intracellular *α*-synuclein (α-Syn) aggregates, are another hallmark and reflect disturbances in protein metabolism and autophagy (Zhang S. et al., 2025). Genetic and environmental factors, α-Syn aggregation, and oxidative stress collectively contribute to PD pathogenesis (Morris et al., 2024). Recent evidence highlights lactate as a key metabolic regulator in PD. Elevated lactate levels have been detected in the cerebrospinal fluid of PD patients, likely arising from impaired mitochondrial function, altered neuronal metabolism, and microcirculatory deficits (Henchcliffe et al., 2008). Mitochondrial complex I (CI) dysfunction reduces oxidative phosphorylation, forcing cells to rely on glycolysis and resulting in lactate accumulation in dopaminergic neurons (Malpartida et al., 2021; Dionisio et al., 2021). Neurotoxic models, including MPTP and rotenone treatment, recapitulate PD-like pathology and similarly induce lactate elevation *in vivo* and *in vitro*. Inhibition of glycolytic enzymes such as hexokinase-2 or pyruvate kinase M2 reduces lactate production, prevents neuronal apoptosis, and improves motor function, demonstrating a causal link between lactate accumulation and PD progression (Li et al., 2022; Lian et al., 2024). Microglial activation is a key mechanism underlying the death of dopaminergic neurons in PD. Lactate injection inhibits neuroinflammation by suppressing classical microglial polarization, indicating that elevated lactate levels possess neuroprotective potential. Conversely, in primary microglia, lactate may enhance the production of pro-inflammatory mediators (Han et al., 2024). In the Parkinson’s disease model, Qin et al. (2025) demonstrated that activated microglia enhance glycolysis, leading to lactate production. Under the catalysis of P300/CBP, lactate drives the lactylation modification of histone H3 at the K9 site, thereby activating Slc7a11 gene expression. The upregulation of Slc7a11 protein exacerbates microglial activation and neuroinflammation, ultimately impairing dopaminergic neurons.

Beyond reflecting metabolic stress, lactate modulates pathogenic pathways in PD. Elevated lactate contributes to α-Syn aggregation, oxidative stress, and neuroinflammation (Komilova et al., 2022; Gonzalez-Rodriguez et al., 2021; Monsorno et al., 2022; Zhang et al., 2022). Lactate-derived histone lactylation mediates epigenetic regulation of neuroinflammatory responses. H3K9 lactylation regulates TNF signaling and microglial activation, while H3K18 lactylation modulates NF-κB signaling and interleukin-6 expression, influencing neuronal survival (He et al., 2024). In PD mouse models, reducing endogenous lactate or directly inhibiting histone lactylation suppresses microglial activation, lowers pro-inflammatory cytokine release, and improves motor outcomes (Qin et al., 2025).

PD’s lactylation pathology is uniquely triggered by substantia nigra dopaminergic neuron mitochondrial CI deficiency, with key targets including mitochondrial protein lactylation and H3K9la-mediated TNF signaling in microglia. This “mitochondrial dysfunction-lactylation-neuroinflammation” cascade is distinct from ischemia’s ANLS disruption or AD’s tau/Aβ-linked lactylation.

Taken together, lactate accumulation and histone lactylation constitute a metabolic–epigenetic axis that links mitochondrial dysfunction, neuroinflammation, and dopaminergic neuron degeneration in PD. Targeting lactate production, glycolytic enzymes, or lactylation pathways may offer novel therapeutic strategies to slow PD progression and mitigate neuroinflammation.

## 8 Discussion

In this review, we synthesize accumulating evidence demonstrating that lactate is not merely a metabolic by-product in the brain, but a central metabolic and signaling molecule whose dysregulation contributes to neurological disease. Across ischemic stroke, AD, and PD, enhanced glycolysis, lactate accumulation, and impaired lactate transport consistently emerge as shared metabolic features. While these observations align with earlier studies emphasizing energy failure and mitochondrial dysfunction in neurological disorders, they extend previous understanding by identifying lactate as an active regulator rather than a passive marker of metabolic stress.

The lactate-lactylation axis exerts disease-specific pathogenic roles via distinct core mechanisms: cerebral ischemia features an acute metabolic crisis-driven lactylation of mitochondrial proteins (Zhou et al., 2024); AD involves chronic neuroinflammation and tau-related lactylation in dual-cell networks (Zhong L. et al., 2023; Zhao and Xu, 2022); PD is characterized by mitochondrial deficiency-linked lactylation in dopaminergic neurons (Han et al., 2024). These disease-specific features, rather than a one-size-fits-all mechanism, confirm the axis’s context-dependent relevance in CNS disorders.

A key contribution of this review is the integration of metabolic and epigenetic perspectives through the concept of protein lactylation. Lactate serves as an efficient oxidative substrate for neurons and participates in neurovascular coupling and memory formation (Mintun et al., 2004; Dienel, 2019). Beyond these established roles, excess lactate can directly influence gene expression and protein function through lysine lactylation, thereby establishing a mechanistic link between altered metabolism and sustained epigenetic regulation (Zhang et al., 2019; Moreno-Yruela et al., 2022). By highlighting lactylation as a metabolic sensor that translates intracellular lactate levels into transcriptional and functional changes, this review provides a unifying framework connecting metabolism, epigenetics, and brain pathology.

Protein lactylation represents a direct molecular mechanism by which metabolic reprogramming affects gene regulation. Elevated intracellular lactate promotes histone and non-histone lactylation, reshaping chromatin accessibility and transcriptional programs involved in inflammation, stress responses, and cell fate determination (Zhang et al., 2019; Moreno-Yruela et al., 2022; Chen et al., 2022). In the central nervous system, neuronal activity–dependent lactate accumulation correlates with increased protein lactylation, indicating that lactylation couples neural excitation to epigenetic control (Hagihara et al., 2021). Disease-specific evidence further supports this framework. In ischemic stroke, lactate accumulation has long been regarded as a consequence of hypoxia-induced glycolysis (Magistretti and Allaman, 2018; Xiong et al., 2024; Weinstein et al., 2004; Berthet et al., 2009; Kalogeris et al., 2012; Zhao et al., 2022). More recent studies demonstrate that lactate-driven protein lactylation actively exacerbates neuronal injury during ischemia, whereas lactate supplementation during reperfusion can exert neuroprotective effects (Xiong et al., 2024). These findings underscore the stage-dependent and context-specific actions of lactate. In AD, the identification of histone lactylation marks such as H4K12la and H3K18la links metabolic dysfunction to sustained microglial activation and inflammatory gene expression, integrating metabolic and epigenetic mechanisms underlying synaptic damage and cognitive decline (Pan et al., 2022; Wei et al., 2023). In PD, mitochondrial CI dysfunction and compensatory glycolysis elevate lactate levels (Morris et al., 2024; Zhang S. et al., 2025), while increased histone lactylation accompanies microglial activation and inflammatory signaling, linking metabolic failure to dopaminergic neurodegeneration (Qin et al., 2025; He et al., 2024).

Collectively, these observations support the working hypothesis that metabolic reprogramming toward glycolysis leads to lactate accumulation, which in turn drives protein lactylation and epigenetic dysregulation contributing to brain disease (Zhang et al., 2019; Moreno-Yruela et al., 2022; Chen et al., 2022; Li et al., 2020). Importantly, lactate and lactylation exert context-dependent effects. Lactate can be neuroprotective when efficiently oxidized or appropriately timed, yet detrimental when chronically accumulated or coupled with excessive lactylation. This duality highlights the importance of cell type, disease stage, and metabolic state when interpreting lactate-related findings.

Notably, similar “metabolic substrate-epigenetic modification” frameworks exist for other metabolites, such as lauric acid, which modulates neural cell redox balance (Ramya et al., 2022) and regulates glucose transport via histone methylation and lncRNA HOTAIR (Ramya et al., 2024), highlighting the conserved nature of this regulatory mode and potential crosstalk with lactate-lactylation.

Despite the compelling evidence linking the lactate–lactylation axis to brain pathology, several critical questions remain. First, the enzymatic machinery governing protein lactylation in neurological disorders—specifically the cell-type-specific “writers” and “erasers”—remains largely unidentified. Determining whether existing HDAC inhibitors or histone acetyltransferase (HAT) modulators exert their effects through the lactylation pathway is a priority for pharmacological research. Second, while this review highlights the duality of lactate, the precise “tipping point” where lactate transitions from a neuroprotective fuel to a pathological epigenetic trigger needs to be defined using high-resolution spatio-temporal mapping. From a therapeutic perspective, the lactate–lactylation axis represents a targetable vulnerability in neurological diseases. Small molecules designed to modulate MCT transporters or site-specific lactylation marks could offer a more nuanced approach than global metabolic inhibition. For instance, selectively blocking lactylation-driven inflammatory programs in microglia while preserving neuronal lactate oxidation could pave the way for precision neurometabolic therapies. Ultimately, deciphering this metabolic–epigenetic code will not only deepen our understanding of brain homeostasis but also provide a new lexicon for treating recalcitrant neurological disorders.

## 9 Conclusion

This review establishes the role of lactate as a critical signaling bridge between cerebral glycolytic reprogramming and epigenetic regulation. Dysregulated lactate metabolism and protein lactylation are now recognized as common features of various neurological disorders, including ischemic stroke, Alzheimer’s disease, and Parkinson’s disease. As a metabolic-epigenetic sensor, lactylation converts intracellular lactate flow into sustained transcriptional programs, thereby regulating neuroinflammation, excitability, and synaptic function. It is noteworthy that the functional effects of the lactate-lactylation axis vary depending on cell type, disease stage, and metabolic state, with disease-specific core mechanisms and targets that avoid overgeneralization of the lactylation pathway. Understanding this biphasic characteristic provides a unified framework for comprehending brain pathological mechanisms and opens a strategic pathway for precision neurotherapy—a therapeutic approach that can both target epigenetic dysregulation and maintain critical energy homeostasis.

## Footnotes

1
http://kla.zbiolab.cn/

2
http://lin-group.cn/server/DeepKla

3
http://tubic.org/Kla
